# p53-Mediated Molecular Control of Autophagy in Tumor Cells

**DOI:** 10.3390/biom8020014

**Published:** 2018-03-21

**Authors:** Maria Mrakovcic, Leopold F. Fröhlich

**Affiliations:** AG VABOS, Department of Cranio-Maxillofacial Surgery, University of Münster, Albert-Schweitzer-Campus 1, 48149 Münster, Germany; maria.mrakovcic@web.de

**Keywords:** p53, mTOR, autophagy, histone deacetylase inhibitor, suberoylanilide hydroxamic acid, tumor

## Abstract

Autophagy is an indispensable mechanism of the eukaryotic cell, facilitating the removal and renewal of cellular components and thereby balancing the cell’s energy consumption and homeostasis. Deregulation of autophagy is now regarded as one of the characteristic key features contributing to the development of tumors. In recent years, the suppression of autophagy in combination with chemotherapeutic treatment has been approached as a novel therapy in cancer treatment. However, depending on the type of cancer and context, interference with the autophagic machinery can either promote or disrupt tumorigenesis. Therefore, disclosure of the major signaling pathways that regulate autophagy and control tumorigenesis is crucial. To date, several tumor suppressor proteins and oncogenes have emerged as eminent regulators of autophagy whose depletion or mutation favor tumor formation. The mammalian cell “janitor” p53 belongs to one of these tumor suppressors that are most commonly mutated in human tumors. Experimental evidence over the last decade convincingly reports that p53 can act as either an activator or an inhibitor of autophagy depending on its subcellular localization and its mode of action. This finding gains particular significance as p53 deficiency or mutant variants of p53 that accumulate in the cytoplasm of tumor cells enable activation of autophagy. Accordingly, we recently identified p53 as a molecular hub that regulates autophagy and apoptosis in histone deacetylase inhibitor-treated uterine sarcoma cells. In light of this novel experimental evidence, in this review, we focus on p53 signaling as a mediator of the autophagic pathway in tumor cells.

## 1. Introduction: Autophagy and Tumorigenesis

Autophagy is a self-degradative process that represents an important physiological catabolic mechanism of the eukaryotic cell. Thereby, organized degradation and recycling of non-functional or non-required cellular components as a reaction to changing conditions is enabled [1,2,3]. The different pathways of autophagy have been classified into three categories: macroautophagy, microautophagy, and chaperone-mediated autophagy [4,5,6,7]. Via lysosomal degradation, basic macroautophagy, to which we refer in this review, allows, in addition to the proteasome-mediated pathway, the turnover of long-lived protein and organelles, the maintenance of anabolic–catabolic homeostasis, the counteraction of aging, and the preservation of energy of the cell. Thereby, autophagy together with apoptosis is also granted a crucial role in cellular quality control [5]. Furthermore, autophagy is particularly indispensable for the cell in its response to nutrient starvation and other types of stressful conditions [8]. Autophagy is encountered during embryonic development and cell differentiation and participates in the innate immune response by eliminating invading intracellular bacteria and viruses. In 2016, the Nobel Prize in Physiology or Medicine was awarded to Yoshinori Ohsumi for his groundbreaking experiments related to the mechanisms of autophagy in starvation-induced non-selective autophagy [1,9,10,11]. Selective autophagy describing the cytoplasm-to-vacuole targeting (CVT) pathway was discovered a few years later by Daniel J. Klionsky´s group [12]. Although the process of autophagy was discovered half a century ago and the term “autophagy” derived from the ancient Greek meaning for “self-eating” was given by the Belgian biochemist Christian de Duve in 1963, its fundamental importance as a physiological cellular mechanism was only appreciated upon Ohsumi’s research in yeast in the 1990s [13]. Subsequently, as autophagy is conserved throughout evolution, the corresponding autophagic machinery involved in its pathway has been discovered in all eukaryotes, including humans [14]. Autophagosomal dysfunction can be caused by genetic mutations that have been associated with the pathogenesis of diseases such as the neurodegenerative Parkinson’s disease, type 2 diabetes, and cancer [15]. Thus, ongoing research is focused on the development of drugs that can target autophagy in these specific diseases. 

The role of autophagy in cancer offers high potential for future therapy and is, therefore, currently also intensively investigated. Different from apoptotic or necrotic programmed cell death, autophagy can pursue either a pro-survival or a pro-death strategy if mediated in tumor cells [15,16]. Specifically, encountered often in apoptosis-resistant tumor cells, autophagy takes on a tumor suppressive function, which limits tumor necrosis and inflammation [17]. In this context, autophagy may be regarded as a protective pro-survival mechanism that inhibits the onset of apoptotic and necrotic cell death in a concerted action [15,18,19,20,21]. Moreover, it can help tumor cells deal with metabolic stress and overcome the cytotoxicity of chemotherapy. In cells and conditions where autophagy may have a supportive function in cell death, however, unelucidated mechanisms seem to expedite the autophagic program [22]. Inhibition of autophagy in tumor cells will, therefore, promote tumor survival. Alternatively, tumor cells could capitalize on autophagy for their survival due to the higher turnover requirements of their metabolism. Regardless of these facts, disruption of autophagy in combination with chemotherapeutic treatment has been approached intensively in cancer therapy. For these reasons there is certainly a lack of knowledge about autophagic signaling in tumor cells. As a consequence, before determining whether autophagy interference can be applied in tumor therapy and a better clinical translation of basic research findings in the future, it is even more important and desirable to first define and gather molecular clues that confirm the context-dependent role of autophagy in tumorigenesis [23,24,25].

## 2. The Cellular Mechanism of Autophagy

During the process of macroautophagy, the controlled formation of a vesicle with a bilayer membrane is initiated allowing the separation of targeted cellular components or organelles from the rest of the cytoplasm [14]. This vesicle, known as an autophagosome, then undergoes a fusion process with lysosomes, which thereby enables degradation and recycling of the engulfed contents by the complemented lysosomal proteolytic enzymes in a low pH milieu [26]. Autophagosomes are formed de novo from the so-called phagophore at the phagophore assembly site, which is also called a pre-autophagosomal structure (PAS) [27]. However, it is still unclear where the phagophore membranes come from. In the current model, it is assumed that the endoplasmic reticulum is the source [28]. This collection of membranes serves as a platform to which the various so-called proteins derived from autophagy-related genes (ATGs) are then recruited. Specifically, Ohsumi´s discovery of ATGs in yeast has tremendously enhanced our understanding of autophagosome formation and led to the isolation of its membrane [29]. These act in a specific sequence, leading to the formation, enlargement, and closure of the autophagosome and have been grouped into the following autophagic stages: initiation (the ATG1/ULK kinase complex), nucleation (the ATG12 conjugation system), elongation (the ATG8/LC3 conjugation/deconjugation system), maturation (the phosphatidylinositol 3-kinase complex), and degradation (the ATG9/ATG9L1 cycling system). At present, more than 20 ATG proteins are known in mammalian cells, contributing to these stages, although the respective functions have not yet been conclusively clarified.

## 3. Autophagosome Formation and Its Molecular Control

**Figure 1:**
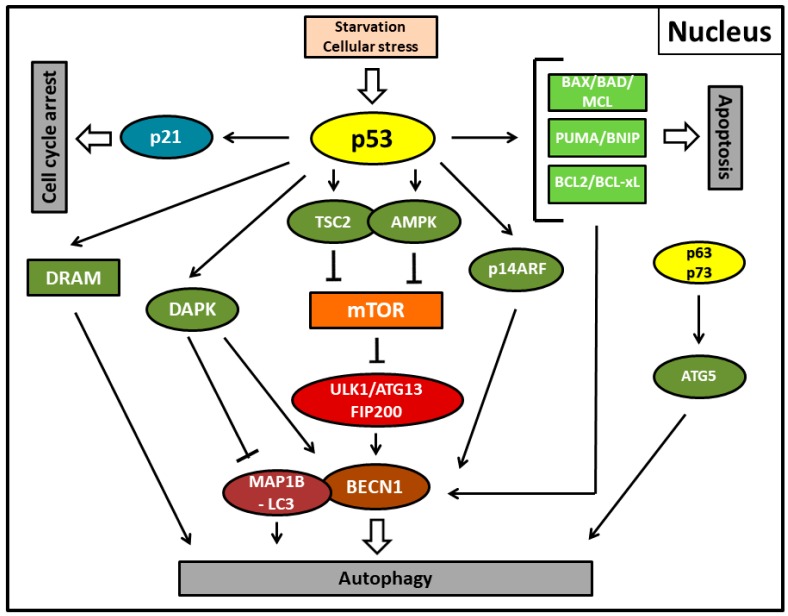
Autophagy, apoptosis, and cell cycle arrest mediated by the activity of nuclear p53 protein (p53) in its function as a transcription factor in stress conditions. p53 primarily induces the canonical pathway of autophagy by transcriptionally upregulating tuberous sclerosis complex 2 (TSC2) (or phosphatase and tensin homolog (PTEN); not shown) and AMP-activated protein kinase (AMPK) (or the AMPK activating sestrins; not shown), thereby suppressing mammalian target of rapamycin (mTOR) and the unc-51-like autophagy activating kinase 1 (ULK1) complex including the autophagy-related protein13 (ATG13) and the focal adhesion kinase interacting protein of 200 kDa (FIP200) further downstream. For ULK1-mediated autophagosome formation or suppression, mTOR interacts further downstream with Beclin-1 (BECN1). Alternatively, damage-regulated autophagy modulator (DRAM), death-associated protein kinase (DAPK), or autophagy-related protein5 (ATG5) upregulation by the p53 family members p63 and p73 are also able to initiate autophagy. DAPK executes autophagy by phosphorylating Beclin-1 or inhibiting LC3-interacting MAP1B protein (MAP1B-LC3). DRAM and p63/p73 can also induce apoptosis. Pro-apoptotic proteins of the Bcl-2 family such as BCL2, BCL-xL, BAX, BAD, MCL, PUMA, and BNIP and the alternate reading frame protein product of the CDKN2A locus (p14ARF) can further induce autophagy via reversal of Beclin-1 inhibition. Fork symbols: inhibition by indicated proteins. Arrow lines: upregulation or activation by indicated proteins. Double arrow: major pathway activity. p53-mediated upregulation of the cyclin-dependent kinase inhibitor 1 (p21) enforces cell cycle arrest.

The molecular control of autophagic activation is dominated by tumor suppressor and oncogene proteins that functionally represent protein kinases [30,31,32,33,34]. Thus, generally, tumor suppressor proteins promote autophagy whereas oncogenes silence this process. Particularly, the nutrient-sensing serine/threonine kinase mammalian target of rapamycin (mTOR), the unc-51-like autophagy activating kinases (ULK1/ ULK2), the Beclin-1 (BECN1) lipid kinase complex, and a ubiquitin-like conjugation system take part in the fine-tuning networks controlling the early stages of autophagosome formation (Figure 1, Table 1) [1,8,35,36,37]. As a common denominator at the molecular level, autophagy is primarily initiated by activation of the mTOR multiprotein complexes (mTORC), which integrate multiple signaling pathways that sensitize genotoxic stress and the levels of reactive oxygen species (ROS), as well as the availability of amino acids, glucose, oxygen, energy, and growth factors [35,38]. Therefore, in addition to autophagy, mTOR regulates numerous other cellular processes, such as cell cycle progression, protein translation, microtubule organization, and lipid biogenesis [39]. By interacting with different proteins, mTOR forms at least two different complexes, mTORC1 and mTORC2, which both include mLST8 and Deptor proteins, in addition to mTOR, but also contain unique components [40]. mTORCs subsequently phosphorylate and further downregulate the activities of a complex containing ATG13, ULK1 (ATG1), and the focal adhesion kinase interacting protein of 200 kDa (FIP200) [41]. This ATG13–ULK–FIP200 complex is required for activating nucleation (i.e., phagophore formation) and is involved in nutrient starvation-induced autophagy [42,43]. However, novel studies also implicate mTOR in the regulation of autophagy via inhibition of p73, a member of the p53 family, which leads to subsequent activation of ATG5, ATG7, and UV radiation resistance-associated gene (UVRAG) [44,45]. For ULK1-mediated autophagic activation, mTOR interacts further downstream with Beclin-1 (ATG6), forming a core complex with Vps15 and class III phosphatidylinositol 3-kinase (PIKC3), also known as Vps34. Activation of PI3KC3 allows the generation of phosphatidylinositol 3-phosphate (PI3P) [37,46]. Thereby, Beclin-1 recruits many proteins to the phagophore and interacts with them in order to coordinately arrange elongation and maturation of the autophagosome or the suppression of autophagy [47]. Nevertheless, Beclin-1 is involved not exclusively in autophagy—as it is phosphorylated itself by the death-associated protein kinase (DAPK)—but in membrane trafficking of lysosomes and endosomes of the cell in general [48]. Beclin-1 is furthermore a known haploinsufficient tumor suppressor protein that is commonly lost in many sporadic tumors of the breast, ovary, and prostate [37]. Final autophagosome maturation requires subsequent recruitment of PI3P-binding proteins WIPI 1/2 and two ubiquitin-like conjugation systems, the ATG12-ATG5-ATG16L and the LC3-phosphatidylethanolamine (LC3-PE) complexes [1,49,50,51]. During the maturation process, cytosolic LC3-I (microtubule-associated protein 1A/1B-light chain 3) is integrated and sequestered to LC3-II. Furthermore, the ubiquitin-binding scaffold protein p62 (also known as SQSTM1) directly interacts and co-localizes with LC3 via a specific sequence motif [52]. Its task may be found in selective autophagy by directing ubiquitinated proteins toward the autophagosome; furthermore, it has been associated with the regulation of deacetylase activity and has been implicated in tumorigenesis [53,54]. Consequently, the protein levels of p62 decline during autophagic induction or accumulate upon autophagic interference. Interestingly, clearance of this autophagic cargo protein was also reported to suppress tumorigenesis [54]. Thus, both LC3 and p62 represent specific markers used for monitoring the autophagic flux [55]. Final fusion with the lysosomal compartment, allowing degradation of the autophagosome content via acid hydrolases and cathepsins, involves the presence of small Rab GTPases and the transmembrane protein LAMP2 [56,57].

## 4. Positive Regulation of p53-Mediated Autophagy

The key tumor suppressor protein p53 has been described, in addition to its numerous other tasks justifying its designation as “gatekeeper of the cell,” as a further important regulator of autophagy. Thus, p53 can react to different kinds of stress and damage exerted on the cell that comprise endogenous- or environmentally-caused oxidative stress, genotoxicity, and oncogene activation in order to prevent cell damage and maintain cellular integrity [58,59]. On the molecular level, activation of p53 is arranged by multiple covalent modifications, including acetylation, methylation, phosphorylation, and ubiquitination [60,61]. Accordingly, as a central transcription factor implementing these posttranslational signals, p53 co-ordinates the expression of genes that control cell-cycle progression, apoptosis, energy metabolism, DNA repair including methylation, and autophagy in a transcription-dependent or -independent fashion [62,63,64,65,66]. Nevertheless, how posttranslational modifications of p53 specify its selectivity for each of these transcriptional targets and the respective cellular programs to induce apoptosis or autophagy is still unclear.

Autophagic regulation by nuclear transactivation—i.e., transcriptional upregulation of its downstream target genes by wild-type p53—in healthy and tumor cells occurs through several pathways in the classical canonical pathway (Figure 1, Table 1) [64]. These include the target genes TSC2 (tuberous sclerosis complex 2) and PTEN (phosphatase and tensin homolog), both of them being determined or presumed tumor suppressors, respectively, as well as the nutrient energy sensor AMP-activated protein kinase (AMPK) or sestrins 1 and 2 that are AMPK activators [67,68,69,70]. Further signaling of the autophagic process by these pro-autophagic factors is then mediated by mTOR inhibition as detailed in the previous chapter [67,71]. As a kind of shortcut and a direct mediator of autophagy and apoptosis, damage-regulated autophagy modulator (DRAM) can be upregulated by stress-activated p53 [72]. DRAM represents a lysosomal protein that can interfere with several different stages of autophagosome formation [73]. In addition, apoptosis-inducing proteins can be directly transactivated by p53 and are also implicated in activation of autophagy [74,75]. Here, downregulating the expression of BCL-2, BCL-xL, and MCL-1 or upregulating the expression of BAX, BAD, BNIP3, or PUMA releases Beclin-1 from its protein complex that initiates autophagy [76].

Via the same mechanism, by directly physically interacting with BCL-xL, the p53-regulated human tumor suppressor protein p14ARF (alternate reading frame protein product of the CDKN2A locus) also seems to be able to induce autophagy [77,86]. The major task of p14ARF was established in the cellular response towards hyperproliferative signals provoking oncogenic activation partially by stabilizing the p53 protein that induces cell growth arrest and apoptosis. However, several groups have reported that human p14ARF can also induce autophagy [86,87,88]. A recent investigation confirmed that by the activation of autophagy, p14ARF also exerts tumor suppressive activity. The same report also untangled previous conflicting results about two p14ARF mRNA isoforms and demonstrated that only full-length p14ARF present in the nucleus can induce autophagy, whereas the small mitochondrial (smARF) isoform induces mitophagy (selective macroautophagy of mitochondria) [89,90]. Moreover, DAPK-1-induced autophagy has been reported to be initiated by p53 via upregulation of its gene expression [48]. DAPK-1 follows two pathways to execute autophagy. One pathway is via the phosphorylation of Beclin-1 as described above, which prevents its degradation by BCL-2/BCL-xL, while the other possibility lies in the inhibition of the anti-autophagic LC3-interacting MAP1B protein [78,79]. Interestingly, DAPK-1-mediated autophagy has been discovered very late since it is not present in yeast.

## 5. Negative Regulation of p53-Mediated Autophagy

**Figure 2:**
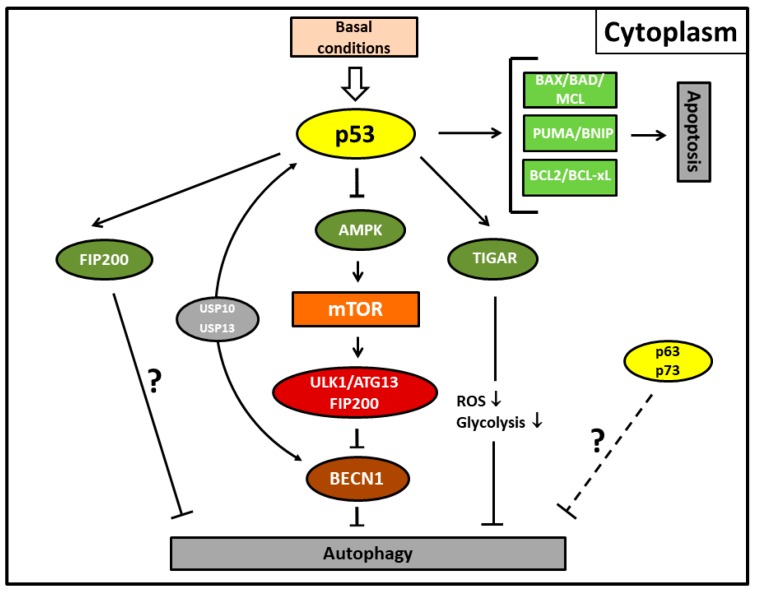
Autophagy and apoptosis mediated by the cytoplasmic activity of p53 protein under basal conditions. Cytoplasmic p53 protein inhibits autophagic cell death by inducing Beclin-1 (BECN1) degradation via the ubiquitin-specific peptidases USP10 and USP13 and/or inhibiting the AMPK-mTOR-ULK1 signaling pathway. It is unclear whether the canonical pathway is mediated by direct p53/FIP200 interaction or whether this represents an extra pathway. TP53-induced glycolysis and apoptosis regulator (TIGAR) inhibits autophagy by downregulation of glycolysis and suppression of reactive oxygen species (ROS) formation. p63/p73 possibly also possess transcription-independent inhibitory functions for autophagy (dashed line). Fork symbols: inhibition by indicated proteins. Arrow lines: activation by indicated proteins; downward arrow, downregulation. For abbreviations, see Figure 1.

In addition to the well-established modulatory functions of nuclear p53 directing the activation of autophagy, key regulatory activities of cytoplasmic p53 protein related to autophagy have been discovered in the last decade (Figure 2, Table 1) [32,64,91,92]. Notably, Tsademir et al. unraveled that depending on its subcellular localization, p53 elicits either pro- or anti-autophagic responses [81]. Thus, stress-induced activation of p53 protein translocated to the cell nucleus is able to stimulate pro-autophagic functions, while physiological p53 protein levels localized in the cytosol have an inhibitory effect on autophagy under normal conditions. This inhibitory effect seems to be independent of its transcriptional function but employs the same canonical AMPK–mTOR signaling pathway cascade as nuclear p53. Thus, in contrast to nuclear p53, the cytoplasm-localized protein inhibits the AMP-dependent kinase, a positive regulator of autophagy, and activates mTOR [81]. As a proof-of-concept, depletion or pharmacological inhibition of basal levels of p53 was found to induce autophagy in vivo and in vitro and this increased autophagy confers resistance to metabolic stress in p53-deficient cells. Furthermore, cells with a genetically modified nuclear export domain in the p53 protein, forcing it to remain in the nucleus, lack suppressive autophagic capabilities and are in line with this conclusion. The exact mechanism of how p53 directly mediates autophagic suppression, which was found to be conserved in nematodes and mammalian cells, needs to be clarified [93]. In this regard, bioinformatic predictions that FIP200 (ATG17) represents at least one interacting partner of p53 in the cytoplasm that regulates autophagy could be verified by mutational analyses [82]. Previous findings demonstrated the molecular link of TIGAR (TP53-induced glycolysis and apoptosis regulator) with the anti-autophagic function of cytoplasmic p53 [83]. As a direct target gene of p53, inhibition of autophagy by TIGAR has been shown to be associated with downregulation of glycolysis and suppression of ROS formation under stressful conditions [94]. If the function of TIGAR is blocked, increased ROS levels initiate induction of autophagy. However, its involvement seems to be unlikely and represents an alternative metabolic pathway as TIGAR does not clearly attenuate mTOR signaling. In embryonal carcinoma cells, cytoplasmic p53 was demonstrated to interact with Beclin-1, which consequently promotes its ubiquitination and degradation and thereby suppresses autophagy [84]. Inactivation of cytoplasmic p53 reverses this effect and promotes induction of autophagy. In an independent study, Beclin-1 could be identified as a mediator of de-ubiquitination activity of p53 by engaging the USP10 and USP13 ubiquitin-specific peptidases [85]. Nevertheless, also in this case, the underlying mechanisms that relate to the nuclear p53-dependent autophagy need to be further elucidated. Beclin-1-induced autophagy mediated by up- or downregulated BCL2-family members can be furthermore blocked by caspase-mediated cleavage of Beclin-1, which simultaneously leads to the production of pro-apoptotic Beclin-1 fragments, triggering apoptosis via cytochrome c release from the mitochondria [95]. This mechanism exemplifies one possibility of bidirectional interaction between apoptosis and autophagy.

## 6. Regulation of Autophagy in p53-Inactivated Cells

In addition to the multifaceted maintenance of cell integrity and homeostasis, the most eminent functions of p53 belong to the supervision of the cell with regard to tumor transformation. This includes the activation of oncogenes, DNA methylation alterations, and genotoxicity [58]. Mutations of the p53 tumor suppressor gene are among the most frequent genetic alterations found in human tumors [96]. Of these, p53 missense mutations with a single amino acid change resulting in the loss of functional wild-type p53 and its tumor suppressor function reflect the prevailing number of cases [97]. In addition, several mutant p53 variants can exert a dominant-negative effect over the remaining wild-type allele or even lead to gain-of-function alleles that carry new oncogenic potential [98,99]. Thus, experimental evidence confirms that tumors with a mutant gain-of-function variant of p53 are characterized by higher genomic instability, reduced chemotherapeutic response, and a generally poor prognosis for patients [100].

Impairment of p53 wild-type function, as provoked by many tumor-derived p53 mutants, can also deregulate autophagy signaling [81,93,101,102]. While the ability of nuclear transactivation-dependent activation of autophagy by p53 is hampered in many cases due to inactivation or lack of p53 in the cytoplasm, the (tumor) cell is able to stimulate pro-autophagic functions. This activity is translated by downregulation of the mTOR complex. Astonishingly, Morselli et al. specified that several tumor-derived variants of p53 with point mutations that steadily localize to the cytoplasm are still able to inhibit autophagy [93]. This finding led to the conclusion that p53 may inhibit autophagy by means of direct protein–protein interactions. Moreover, it demonstrates the importance to discriminate p53 variants with respect to null or point mutations, keeping in mind that nonsense mutations can also lead to degradation of the p53 transcript. Although inactivated p53 seems to be counterproductive with regard to the tumor suppressor function of p53, it makes sense when considering that inactivated autophagy by mutant p53 enforces cell survival. Consistent with this idea, a study has confirmed that gain-of-function mutant p53 proteins inhibit the autophagic pathway and thereby enhance the proliferation of pancreas and breast cancer cells. This counteractivity is accompanied by repression of Beclin-1, DRAM, ATG12, and sestrin genes, as well as stimulation of AMPK–mTOR genes [103]. Several studies have demonstrated the mutual functional surveillance between autophagy and p53 in the cell. Thus, on the one hand, mutant p53 suppresses autophagy while on the other hand autophagy can trigger the degradation of mutant p53 in order to prevent tumorigenesis. On the molecular level, this counter-surveillance mechanism is implemented by the reciprocal crosstalk of the two suppressor genes Beclin-1 and p53 with regard to the regulation of autophagy, as outlined above [85]. By regulating its de-ubiquitination, Beclin-1 directly controls the protein levels of p53, which is likely also reflected by the phenotype of Beclin-1-deficient mice that mirror the ablated functions of the tumor suppressor p53 [104,105]. Related to Beclin-1 mediated autophagy, the tumor suppressor protein p14ARF was also found to be upregulated in p53-silenced or –inhibited cells, as well as in tumor cells and was able to activate autophagy [87,88,89]. Vice versa, overexpression of p14ARF could suppress the proliferation of p53-null cells [88]. However, several additional unexplored mechanisms must exist for p14ARF-elicited autophagy as only a small fraction of its protein interacts with the BCL-xL/Beclin-1 complex.

Interestingly, a study that analyzed the regulation of autophagy in doxorubicin-treated p53-proficient (p53+/+) or -deficient (p53−/−) mouse embryonic fibroblasts found that the p53 family members p63 and p73 are able to compensate for the loss of p53 [80,106]. Doxorubicin is an antitumor antibiotic medication that is frequently used for chemotherapeutic treatment of many different cancers. By chromatin immunoprecipitation sequencing analyses, it was unveiled that, in response to doxorubicin and different stress stimuli, p53 family members translocate to the nucleus and transcriptionally activate an extensive network of ATGs, such as ATG4a, ATG4c, ULK1, ULK2, and UVRAG, as well as ATG5. In conclusion, basic p63 and p73 protein levels could possibly also replace the inhibitory role of wild-type p53 in the cytoplasm of p53-deficient cells but this scenario could be prevented by dominant-negative effects of mutant p53 proteins. Moreover, it was determined that resuming autophagy by p63 or p73 is essential for efficient tumor suppression and prevention of cell transformation. 

## 7. HDACi-Induced Autophagy Mediated by p53

Histone deacetylase (HDAC) inhibitors (HDACi) are a highly investigated class of anticancer therapeutic agents with encouraging clinical activity against hematologic and solid tumors [107,108]. Their inhibitory effect on histone deacetylation is associated with chromatin relaxation and re-expression of silenced genes including non-histone proteins, such as p53 [109]. Thus, it could be demonstrated that HDACi, which prevent HDAC1 deacetylation, promote p53-induced transcription [110]. Thereby, the important cellular tumor-suppressive activities such as regulation of cell cycle arrest, differentiation, and cell death or the suppression of oncogenes can be resumed [111,112]. Moreover, although the precise mechanisms of HDACi are still being elucidated by protein acetylation posttranslational modification, the protein’s stability, proteasomal degradation, cellular distribution including nuclear export, coactivator recruitment, and molecular interaction can be modified [113,114,115,116]. For p53, several acetylation sites have been determined that either augment DNA binding or cause a loss of transcriptional activity; for example, loss of p53-dependent p21 transcription can be caused by deletion of the C-terminal acetylation residues [60,117,118,119].

Up to now, induction of caspase-induced apoptosis was determined as the preferred mode of HDACi-mediated cell death engaging either the extrinsic or intrinsic pathway mostly by activating death receptors or via their ability to stimulate ROS production, respectively [120,121,122]. Nevertheless, a growing number of publications implies that HDACi, such as suberoylanilide hydroxamic acid (SAHA), can alternatively or additionally activate autophagy as an anti-tumor response [123,124,125,126,127,128,129,130,131,132]. In the case of SAHA-induced autophagy, even a pro-survival mechanism could be verified that opposes SAHA-mediated cytotoxicity and comes along with a delayed onset of apoptosis in tumor cells [133,134]. As apoptosis resistance is encountered frequently, HDACi-triggered autophagy would offer a favorable alternative possibility to eliminate tumor cells on the one hand while, on the other hand, SAHA-induced apoptosis could also be efficiently enhanced by genetically/pharmacologically blocking autophagy (e.g., chloroquine or 3-methyladenine) [133,135,136]. Consequently, lysosomal integrity, cytosolic accumulation of cathepsin D, reduced expression of TRX, and high levels of ROS generation were found. Hence, HDACi-induced autophagy was concluded to be an unpleasant consequence of their mechanism of action. 

As persistent overexpression of the HDAC class II enzyme HDAC2 in malignant endometrial stromal sarcoma was identified in a previous study, we aimed to clarify the therapeutic options of the HDACi SAHA for therapeutic treatment of this tumor [137]. By using two different uterine sarcoma cell lines as models in vitro, the cytotoxic efficiency of SAHA treatment was evaluated experimentally. It resulted in cell cycle arrest at the G1/S transition, significantly accelerated cell death with associated increased levels of p21, and reduced expression of HDAC2 and 7 [124,138,139]. In the course of their closer molecular evaluation, different modes of cell death were unveiled in these cells in response to SAHA. Either predominant autophagy in the case of endometrial stroma sarcoma-derived ESS-1 cells or prevailing apoptosis in the case of carcinosarcoma-derived MES-SA cells were observed in vitro and in xenografted tumors. Notably, in ESS-1 cells, SAHA-mediated dose-dependent autophagic cell death was associated with attenuation of mTOR expression [102,124]. However, the question remained which signaling pathways initiate SAHA-induced autophagy upstream of mTOR. Previous publications in this context investigating cancer cell survival under nutrient deprivation observed the transcriptional upregulation of LC3 by SAHA and its downregulation by p53 [126,140]. However, autophagy cannot be activated merely by LC3. An explanation could be provided, however, by the regulation of non-histone proteins, e.g., transcription factors via HDACi-mediated acetylation that indirectly control mTOR-mediated autophagy. 

By examining the key regulators of apoptosis and autophagy upstream of mTOR, the lack of p53 protein and decreased levels of PUMA (p53 upregulated modulator of apoptosis) expression were presumed to be caused by an R213X nonsense mutation located in the transactivating domain of p53 in ESS-1 cells [102]. Subsequently, we were able to overcome apoptosis resistance by rescuing p53-deficiency. Consequently, the prevailing apoptotic cell death was testified for these cells by increased PUMA and caspase-9 expression, activation of caspases-3 and -7, and PARP-1 cleavage. Concurrent basic autophagy was confirmed by raised mTOR expression, leading to decreased autophagosome formation as indicated by LC3 and MDC staining. As a proof-of-concept, several other p53-deficient tumor cell lines undergoing SAHA-induced autophagy were employed in which apoptosis resistance could also be reactivated.

**Figure 3:**
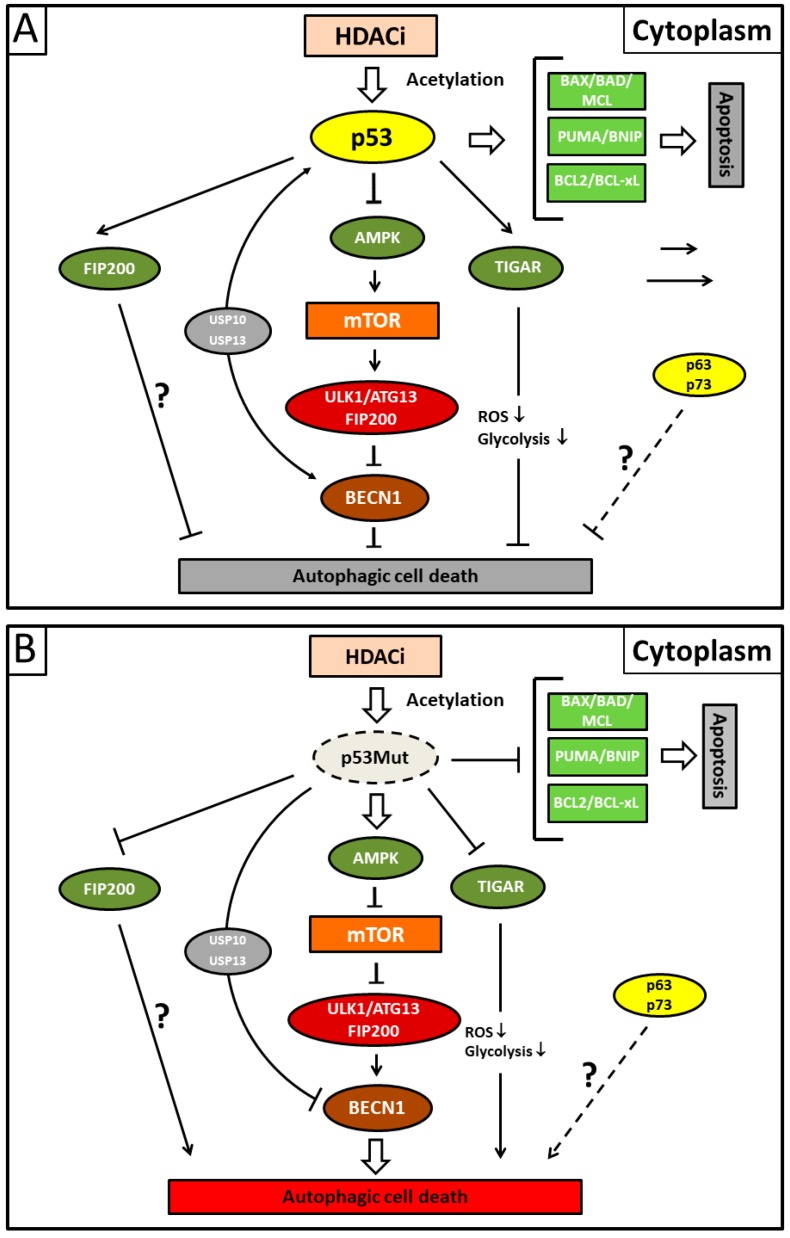
Illustration showing presumed mechanisms mediating suberoylanilide hydroxamic acid (SAHA)-induced autophagy. (**A**) Acetylated cytoplasmic p53 protein that escapes deacetylating activity by applying HDACi (histone deacetylase inhibitor) preferentially induces apoptotic cell death by direct interaction with the Bcl-2 family of pro-apoptotic proteins. Concurrently, cytoplasmic p53 protein inhibits autophagic cell death by inducing Beclin-1 degradation via the ubiquitin-specific peptidases USP10 and USP13 and/or inhibiting the AMPK-mTOR-ULK1 signaling pathway. It is unclear whether the canonical pathway is mediated by direct p53/FIP200 interaction or whether this represents an extra pathway. TP53-induced glycolysis and apoptosis regulator (TIGAR) inhibits autophagy by downregulation of glycolysis and a suppression of ROS formation. The p53 family members p63/p73 possibly also possess transcription-independent inhibitory functions for autophagy (dashed line). (**B**) Mutant p53 (p53Mut) protein reverses the situation and predominantly activates autophagy due to its inability to inhibit autophagy or stimulate apoptosis. Fork symbols: inhibition by indicated proteins. Arrow lines: activation or interaction with indicated proteins. Double arrow: major pathway activity. For abbreviations, see Figure 1.

In conclusion, these findings point to a master regulatory role for p53 with regard to SAHA- and maybe also HDACi-mediated autophagic and apoptotic cell death in general (Figure 3) [132,141,142]. Consequently, p53 deficiency could provide an explanation for both apoptosis resistance and the prevailing SAHA-induced autophagy in tumor cells. The suggested inhibitory role for the functional wild-type p53 protein in SAHA-mediated autophagy is, furthermore, highly consistent with the previous report by Tasdemir et al., indicating the additional crucial role of cytoplasmic p53 as a central coordinator of autophagy that we discussed in the previous sections [81]. In summary, nuclear p53 protein facilitates autophagy while cytoplasmic p53 protein silences it. Thus, our data could give important information about how SAHA treatment is connected to the mTOR signaling pathway, which has been less explored so far. Nevertheless, future experiments need to address the exact underlying molecular mechanisms that were found in the direct interference of SAHA with HDAC activity responsible for deacetylating the non-histone protein p53. This idea can be supported by a previous publication reporting about modified apoptosis in HepG2 cells by SAHA-induced acetylation of p53 [143].

This study highlights again the need to address the context-specific function of the oncogenic tumor suppressor p53 in promoting or impeding autophagy before tumor therapy should be applied. In support of our study, increased acetylation of p53 by sirtinol was also identified as a molecular cause of autophagic cell death besides other antiproliferative effects, such as cell cycle arrest and apoptosis, in the breast cancer cell line MCF-7 [135]. Sirtinol is a specific inhibitor of the class III NAD-dependent deacetylases SIRT1 and SIRT2 that execute essential functions in the regulation of mitosis, survival, and senescence, and are known to target p53. In addition to increased acridine orange and MDC staining, augmented expression of the autophagy marker LC3-II could also be detected after sirtinol treatment of MCF-7 cells. Apoptosis induction that was attributed to increased autophagy induction by the presence of p53 was documented by increased BAX expression, by decreased BCL-2 protein, and by cytochrome c release. Inhibition of autophagy by 3-methyladenine led to an increase in apoptotic cell death in these cells, strengthening the idea that p53 acts as a regulator of HDACi-induced cell fate.

A similar study investigated the anticancer effects of MHY2256, a further potent inhibitor of SIRT1 enzymatic activity as well as SIRT1, 2 and 3 expression levels in MCF-7 cells [144]. The results indicated that inhibition of SIRT1 by MHY2256 causes increased acetylation of p53 at lysine 382. Consequently, increased p53 levels provoked attenuated p53 degradation due to increased expression levels of the ubiquitin ligase MDM2, an important negative regulator of p53. A role for Sirt1 in the regulation of autophagy has subsequently been demonstrated more directly by increasing its expression transiently and a transfer of the Sirt1 wild-type gene into mouse embryonic fibroblasts with a homozygous Sirt1 deletion [145]. In contrast, restoration of autophagy under starvation conditions of these cells was not possible with a deacetylase-inactive mutant of Sirt1. As possible targets of Sirt1-mediated deacetylation activity, the autophagy-related proteins Atg5, Atg7, and Atg8 were found to be significantly acetylated in the absence of Sirt1.

## 8. Conclusions and Outlook

Autophagy is a basic process that is essential for normal cellular activity as its deregulation is commonly encountered during the development of human tumors. Nevertheless, autophagy can be compared to a two-edged sword and possesses an ambiguous role in tumor progression. Depending on the tumor entity and the molecular predisposition in terms of tumor mutations, it can either promote or suppress tumorigenesis [16,31,146,147]. One important task of autophagy describing a tumor-suppressive function lies in the prevention of genomic instability and genotoxic stress leading to DNA damage [148]. The associated oncogenic transformation of the cell is precluded by the disposal of damaged organelles and proteins that represent major sources of ROS. As a representative example besides mutant p53 protein, autophagy regulates cellular levels of the p62/SQSTM1 protein that is frequently mutated in human cancer, as well as upregulated in RAS oncogene-transformed cells, and thereby suppresses the development of tumors [54]. By enhancing metabolic cellular reorganization of transformed cells, however, autophagy supports tumor cells such as cancer stem cells to overcome stressful conditions and promotes tumor survival [149,150]. Consistent reports suggest that autophagy is a necessary step for establishing tumor formation during metabolic stress [151,152]. This tumor-stimulating role of autophagy has triggered investigations to explore the effects of cytotoxic agents in causing therapeutic resistance. Different studies have reported that either pharmacological inhibition (e.g., by chloroquine or 3-methyladenine) of autophagy or genetic inactivation of regulatory autophagy genes sensitize tumor cells to cell death induction upon treatment with diverse compounds [153]. As autophagy is under the stimulatory control of tumor suppressor networks and suppressive effects of oncogenes, the fact that especially these regulator molecules are frequently mutated is of significant importance in this context [32]. p53 is one possible checkpoint residing at the top of these tumor suppressor cascades that controls autophagy even before mTOR and Beclin-1 complexes come into play.

In this review, we outlined the remarkable dual function of p53 in controlling autophagy and eliciting an increased autophagic response by both transcriptional activation and deficiency of p53 [64]. Thus, on the one hand, nuclear transcriptional activity of wild-type p53 activates several pro-autophagic target genes in response to stress stimuli, such as oncogenic activation. On the other hand, it was found that endogenous cytoplasmic p53 represses autophagy that is reactivated upon depletion of a functional p53 protein. According to our recent finding of a genetic nonsense mutation that causes a homozygous loss of p53 expression in uterine sarcoma cells, we concluded that p53 could also represent a “molecular crossing” with regard to HDACi treatment in tumor cells [102]. This seems plausible as non-histone proteins, such as p53, are direct targets of HDACs that are regulated by HDACi whose interference with autophagy has been demonstrated previously. Thus, depending on the mutational status of the tumor cell, HDACi treatment could provoke either autophagy in the absence of the p53 protein or apoptosis in tumor cells harboring wild-type p53 protein. These considerations gain particular importance when deciding about possible cancer therapeutics and the anticipation of a potential clinical outcome in different types of tumors. Nevertheless, a clear-cut strategy awaits further direct experimental evidence in this respect. Further research will also increase our understanding of the principal molecular mechanisms of p53 that are involved in modulating autophagy and apoptosis.

## Figures and Tables

**Table 1:** Different mechanisms of p53-mediated regulation of autophagy.

p53-Induced Autophagy	Molecular Mechanism	Additional Mechanism	Ref.
Activation	TSC2 upregulation	mTOR inhibition	[68]
	AMPK/PTEN upregulation	mTOR inhibition	[69]
	Sestrin 1 and 2 upregulation	mTOR inhibition	[70]
	DRAM upregulation	Direct autophagosome formation	[73]
	Downregulation of BCL-2, BCL-xL, MCL-1	Release of BECN1	[76]
	Upregulation of BAX, BAD, BNIP3, PUMA	Release of BECN1	[76]
	p14ARF upregulation	BCL-xL mediated BECN1 release	[77]
	DAPK-1 upregulation	Release of BECN1 by phosphorylation	[78]
	DAPK-1 upregulation	Inhibition of MAP1B-LC3	[79]
	p63 and p73 upregulation	ATG5, ATG7, UVRAG upregulation	[45,80]
Inhibition	AMPK inhibition	mTOR activation	[81]
	FIP200 interaction	?	[82]
	TIGAR upregulation	Downregulation of glycolysis, suppression of ROS	[83]
	BECN1 interaction	BECN1 ubiquitination and degradation	[84]
	BECN1 interaction	De-ubiquitination of p53 via USP10 and USP13	[85]
